# Sustainable Release of Propranolol Hydrochloride Laden with Biconjugated-Ufasomes Chitosan Hydrogel Attenuates Cisplatin-Induced Sciatic Nerve Damage in In Vitro/In Vivo Evaluation

**DOI:** 10.3390/pharmaceutics14081536

**Published:** 2022-07-23

**Authors:** Yasmin M. Ahmed, Raha Orfali, Doaa S. Hamad, Mostafa E. Rateb, Hanan O. Farouk

**Affiliations:** 1 Department of Pharmacology and Toxicology, Faculty of Pharmacy, Nahda University, Beni-Suef 62521, Egypt; yasmain.mostafa@nub.edu.eg; 2 Department of Pharmacognosy, College of Pharmacy, King Saud University, P.O. Box 2457, Riyadh 11451, Saudi Arabia; 3 Department of Pharmaceutics, Faculty of Pharmacy, Nahda University, Beni-Suef 62521, Egypt; doaa.saad@nub.edu.eg (D.S.H.); hanan.osman@nub.edu.eg (H.O.F.); 4 School of Computing, Engineering and Physical Sciences, University of the West of Scotland, Paisley PA1 2BE, UK

**Keywords:** propranolol HCl, surface modification, chitosan–ufasomes, sciatic nerve, cisplatin

## Abstract

Peripheral nerve injuries significantly impact patients’ quality of life and poor functional recovery. Chitosan–ufasomes (CTS–UFAs) exhibit biomimetic features, making them a viable choice for developing novel transdermal delivery for neural repair. This study aimed to investigate the role of CTS–UFAs loaded with the propranolol HCl (PRO) as a model drug in enhancing sciatica in cisplatin-induced sciatic nerve damage in rats. Hence, PRO–UFAs were primed, embedding either span 20 or 60 together with oleic acid and cholesterol using a thin-film hydration process based on full factorial design (2^4^). The influence of formulation factors on UFAs’ physicochemical characteristics and the optimum formulation selection were investigated using Design-Expert^®^ software. Based on the optimal UFA formulation, PRO–CTS–UFAs were constructed and characterized using transmission electron microscopy, stability studies, and ex vivo permeation. In vivo trials on rats with a sciatic nerve injury tested the efficacy of PRO–CTS–UFA and PRO–UFA transdermal hydrogels, PRO solution, compared to normal rats. Additionally, oxidative stress and specific apoptotic biomarkers were assessed, supported by a sciatic nerve histopathological study. PRO–UFAs and PRO–CTS–UFAs disclosed entrapment efficiency of 82.72 ± 2.33% and 85.32 ± 2.65%, a particle size of 317.22 ± 6.43 and 336.12 ± 4.9 nm, ζ potential of −62.06 ± 0.07 and 65.24 ± 0.10 mV, and accumulatively released 70.95 ± 8.14% and 64.03 ± 1.9% PRO within 6 h, respectively. Moreover, PRO–CTS–UFAs significantly restored sciatic nerve structure, inhibited the cisplatin-dependent increase in peripheral myelin 22 gene expression and MDA levels, and further re-established sciatic nerve GSH and CAT content. Furthermore, they elicited MBP re-expression, BCL-2 mild expression, and inhibited TNF-α expression. Briefly, our findings proposed that CTS–UFAs are promising to enhance PRO transdermal delivery to manage sciatic nerve damage.

## 1. Introduction

There are 43 motor and sensory nerves that connect the CNS to the PNS. The skull and vertebrae protect the CNS, but not the PNS [1]. Peripheral nerve injury (PNI) affects more than one million individuals annually, making it a global clinical issue with a substantial socioeconomic cost [2]. Various factors, including autoimmune illnesses, infections, and trauma, can lead to PNI [3]. PNI is the deterioration of the peripheral nerve structure leading to the loss of deep tendon reflex, sensory nerve dysfunction, and motor and muscle weakness [4]. Axonal degeneration, segmental demyelination, or both may occur in the afflicted nerves. In addition, peripheral neuropathy can result in excessive myelin and axon loss [5]. Toxic chemotherapeutic agents are one of the forms of PNI [6]. Cisplatin, the most widely used anticancer medication, frequently generates peripheral neuropathy [7,8]. Most patients treated with cisplatin experience chemotherapy-induced peripheral neuropathy as a common adverse reaction that reduces the effectiveness of treatment and decreases a patient’s chance of survival as a side effect [9,10].

Cisplatin generates dose-dependent peripheral neuron destruction or ototoxicity [11]. The prolonged cisplatin treatment affects the major cisplatin target, the dorsal root ganglia (DRG), causing sensory and motor neuron loss [12,13]. Neuropathy has been associated with mitochondrial DNA damage [14,15]. The degree of injury is demonstrated through binding cisplatin to DNA in DRG neurons with a high tendency for platinum adduct formation [7,16]. On the other hand, current research suggests that neural axon damage caused by cisplatin may be associated with the suppression of autophagy and mitophagy, resulting in the accumulation of oxidative damage in proteins and organelles [17,18]. Early axonopathy is probably caused by a mechanism involving the formation of nuclear DNA-Pt adducts and mitochondrial DNA-Pt adducts [19], resulting in the production of oxidative stress [20]: inflammatory and pro-inflammatory cytokines [21]. Potentiating nerve injury causes a direct change in bone–nerve interaction and loss of bone mineral density (BMD) [22,23]. Direct effects of nerve injury on BMD are difficult to prove since the sciatic nerve constriction causes immobilization, paralysis, and a decrease in mechanical loading, all of which reduce bone mineral density [5,24,25].

When deciding on an appropriate treatment strategy for PNI, the injury type and its extent are considered [26]. An end-to-end suture is commonly utilized for injuries with small gaps (less than 5 mm), while an autologous nerve graft is ideal for more significant gaps [27,28]. Autologous nerve grafts have various drawbacks, including donor site morbidity, tissue scarcity, and infection risk [29]. These limits have led to neural scaffolds that support nerve cell growth and transport various nerve medicines.

Different scaffolds are utilized in neural regeneration [30]. Hydrogels are popular among these materials due to their three-dimensional structure analogous to nerve tissue and their physicochemical and biological properties [3,31]. In addition, hydrogels possess appropriate physical and biological qualities, such as the capability to absorb water, a similarity to the extracellular matrix (ECM) of nerve, and a porous structure, which makes them excellent candidates in the field of neural tissue engineering [32,33,34,35]. Hydrogel can be made using synthetic or natural polymers. However, natural polymers are preferred due to their biocompatibility and lower costs [36,37]. Chitosan (CTS) is a non-cytotoxic, biodegradable, naturally occurring polysaccharide proposed to assist nerve regeneration in the PNS [38,39]. It has been widely employed in gene delivery [40,41], cell culture [42,43], and biomedical engineering [44]. In addition, CTS also has anti-inflammatory effects on the influx of neutrophils into organs, levels of tumor necrosis factor-alpha (TNF-α), levels of interleukin-1 beta (IL-1), and anti-oxidative properties [44]. Additionally, the porous structure of CTS and hydrogel allows them to carry medicines effectively, encouraging the proliferation of neural stem cells [45].

An effective tissue engineering construction should include a bioactive agent capable of promoting healing in addition to structural support [46]. Adrenoceptor blocker medications have been utilized in neural tissue engineering to enhance the recovery of the nervous system’s function after a traumatic injury. Propranolol hydrochloride (PRO) is a nonselective β-adrenoreceptor blocker widely used to treat hypertension. PRO could regulate numerous pathological conditions, such as cardiac contractions and relaxations [47] and many immunomodulatory, anti-inflammatory, and antioxidant effects [48]. Propranolol coupled with a Gi-coupled receptor protein leads to decreased cAMP [49]. Calcium influx reduction toward cells blocks vascular endothelial growth factors, lowering vasoconstriction and angiogenesis [50]. Additionally, it down-regulates apoptosis of hemangioma-derived stem cells or pericytes in endothelial cells via down-regulation of CDKN1B, AKT, and angiotensin II [51,52].

On the other hand, the anti-inflammatory properties of propranolol reduce local and systemic inflammation, helping the body heal faster while preventing cellular damage, collagen deposition, and the activity of matrix metalloproteases [47,53]. Several studies reveal the role of sympathetic innervation in modulating bone resorption and bone cell activity [54,55]. Additionally, β-blockers induced trabecular bone volume in a mice ovariectomized model [56,57]. A double-blind human model found that the propranolol-treated group experienced considerable bone mass recovery [58].

Despite its significant pharmacological potential, propranolol HCl (PRO) utilization was restricted due to its hydrophilic nature, poor oral bioavailability (15–23%), and extensive first-pass hepatic metabolism [59]. As a result, it is imperative to find new ways to improve the skin permeability of PROs; addressing the obstacles above is a crucial priority in tailoring formulations for clinical use.

Interestingly, various delivery systems were suggested to enhance PRO skin permeation, including polymeric film [60], iontophoresis [61], transethosomes [62], and nanoparticles [63]. Herein, PRO skin permeability was improved with the development of ufasomes (UFAs), which are non-phospholipid vesicles. UFAs were initially developed by Gebicki and Hicks [64] as “unsaturated fatty acid vesicles” with a closed lipid bilayer membrane. They belong to fatty acid vesicles that comprise fatty acid and their ionized species [65,66]. The primary components of UFAs are typically unsaturated fatty acids such as oleic acid and linoleic acid, and their use has several benefits. Due to single-chain amphiphiles, UFAs have a more dynamic nature than their well-known precursor liposomes. They are more versatile by positioning them between traditional double-chain amphiphiles’ nanosystems and micelles [65]. Moreover, UFAs are distinguished by their biocompatibility and straightforward assembling method [66]. Their implementation was previously described in accentuating topical delivery of fluconazole [67] and transdermal delivery of clotrimazole for antifungal activity [68]. Only the current study scrutinizes this novel nano-cargo prospective for PRO anti-sciatic nerve activity.

This study developed a CTS–UFA hydrogel loaded with PRO to investigate its potential for improving sciatic nerve regeneration. Adopting full factorial design, the effect of UFA’s formulation variables on drug entrapment, zeta potential, particle size, PDI, and the in vitro release were inspected. Then, PRO–CTS–UFA was assembled and characterized using a transmission electron microscope, stability study, and ex vivo permeability studies compared to optimal PRO-UFA. In addition, the in vivo study investigated the potential protective benefits of PRO–CTS–UFA gel, compared to PRO solution and PRO–CTS–UFA gel, on rats subjected to cisplatin-induced sciatic nerve injury. To fulfill this purpose, the specific nerve injury biomarker peripheral myelin 22 gene expression, inflammatory biomarker (CAT), and the oxidative stress biomarkers MDA and GSH were analyzed in tissue. Additionally, the immunohistochemical study of MBP, BCL-2, and TNF-α coupled with a histopathological examination of the sciatic nerve section was conducted.

## 2. Materials and Methods

### 2.1. Materials

Propranolol hydrochloride, cholesterol, and sorbitan monostearate (span 60) were obtained from Sigma-Aldrich (St. Louis, MO, USA), while span 20 was obtained from Atlas chemical industries, Wilmington, DE. Oleic acid, Na_2_HPO_4_, KH_2_PO_4_, KCl, and NaCl were purchased from El-Nasr Chemical Co (Cairo, Egypt). Absolute methyl alcohol (99%) and chloroform were purchased from United Company for Chemical preparations (Cairo, Egypt). Dialysis bags with 12000 Da molecular weight cut-off were purchased from SERVA Electrophoresis GmbH (Heidelberg, Germany). Cisplatin (catalog number 15663-27-1) was purchased from Sigma chemicals (Saint Louis, MO, USA). Other products used include enzyme-linked immunosorbent assay (ELISA) kits for MDA (catalog number CSB-E08558r; CUSABIO, Houston, TX, USA), GSH (catalog number MBS724319; MY BIOSOURCE; San Diego, CA, USA), CAT (catalog number CSB-E13439r; CUSABIO, Houston, TX, USA), and PCR gene expression assay for Peripheral myelin 22 (catalog number R2072, Zymo Research Corp., Irvine, CA, USA). Other chemicals and solvents were of analytical grade and used without modifications.

### 2.2. Fabrication of PRO–UFAs

PRO–UFAs were prepared using thin-film hydration methods as labeled by Al-Mahallawi et al. [69], with a minor alteration. In a nutshell, the measured quantity of 10 mg PRO and a definite amount of oleic acid, span, and cholesterol were dissolved in a 10 mL chloroform/methanol (2:1 *v*/*v*) combination. A thin, dry layer was created on the flask wall using a rotary evaporator (Heidolph Laborota 4000 Series, Heizbad, Germany), spinning at 60 rpm and 60 °C in vacuum. Then, hydration of the produced lipid film with phosphate buffer saline (PBS, 10 mL, pH 7.4) was performed via rotating the flask in a water bath at 60 °C for 30 min. The size of the resulting vesicles was through sonication for 10 min in a bath sonicator (Sonix TV ss-series, North Charleston, SC, USA) [70]. The constructed nanodispersions were refrigerated overnight at 4 °C for maturation.

### 2.3. Characterization and Optimization of PRO–UFAs

#### 2.3.1. Determination of PRO Entrapment Efficiency Percent (EE%)

By subtracting the amount of non-entrapped PRO (free PRO) from the amount of PRO initially added, the amount of PRO held within the formulated preparation was indirectly estimated as 10 mg [71]. Briefly, dispersions of PRO–UFA were centrifuged at 16,500× *g* for two hours at 4 °C (Laborzentrifugen, Sigma, Osterode, Germany). The concentration of PRO was evaluated spectrophotometrically (Shimadzu UV-1800, Tokyo, Japan) by measuring the UV absorbance at λ_max_ 290 nm. The EE% of PRO was computed as follows:

(1) $$\mathrm{EE}\%=\frac{\mathrm{Total}\;\mathrm{drug}\;\mathrm{concentration}-\mathrm{free}\;\mathrm{drug}\;\mathrm{concentration}}{\mathrm{Total}\;\mathrm{drug}\;\mathrm{concentration}}\times 100$$

#### 2.3.2. Particle Size (PS), Zeta Potential (ZP), and Polydispersity Index (PDI) Determination

Using Zetasizer Nano 7.11 (Malvern Instruments, Malvern, UK) and the dynamic light-scattering method at 25 °C and a 90° incident beam angle, the average PS, ZP, and PDI of PRO–UFAs were measured. Before the measurements, 0.1 mL of each dispersion was diluted with 10 mL of deionized water to confirm that the intensity of light scattering was within the sensitivity range of the instrument. All measurements were taken in triplicate, and the obtained mean values were recorded [72].

#### 2.3.3. In Vitro Release Study of PRO–UFAs

Using Erweka DT-720 USP type 1 dissolution test (Heusenstamm, Germany), the membrane diffusion method [73,74] was utilized to assess the PRO release from the produced UFAs in triplicate. As determined by the calculated EE%, accurate aliquots of PRO–UFAs (equal to 3 mg of PRO) were injected with the pre-impregnated dialysis membrane (Mol. Wt. cut off = 12,000 Da) covering one end of the glass cylinders (2.5 cm internal diameter and 6 cm length). USP dissolution tester shafts were fitted with glass cylinders containing the loaded samples. The glass cylinder was then submerged in 50 mL of the releasing medium (PBS, pH 5.5). The rotation was adjusted to 100 rpm, and the temperature was kept at 37 ± 0.5 °C for the experiment. The entire volume of the release medium was replaced with an equal volume of the new release medium at predetermined intervals (0.25, 0.5, 1, 2, 3, 4, 5, and 6 h). The release profiles were compared to the PRO solution and PRO–UFAs. The proportion of PRO released was computed based on the total amount of drug released at each interval. By obtaining UV absorbance at 290 nm, drug concentration was measured. All measurements were conducted in triplicate, and results were expressed as the mean of three runs (mean) standard deviation (SD).

Using several kinetic equations, the release behavior of PRO–UFAs was kinetically analyzed. The results were fitted into various mathematical equations to study the release data, including zero- and first-order kinetics, Higuchi, Korsmeyer–Peppas, and Hixson–Crowell models. For each model, the correlation coefficient (R^2^) was calculated.

#### 2.3.4. Studying the Impact of Formulation Variables Using Full Factorial Design

The characteristics of PRO–UFA dispersions were assessed and optimized using a 2^4^ full factorial design. In the design utilized, four factors were estimated, which are (A: type of span), (B: the amount of oleic acid), (C: the amount of cholesterol), and (D: sonication time), represented by low (−1) and high (+1), respectively, Table 1. The experiments were achieved with all possible combinations for fabricating PRO–UFAs formulation, as shown in Table 2. The EE% (Y1), PS (Y2), PDI (Y3), ZP (Y4), and cumulative % PRO released from UFAs in 6 h (Q6h) (Y5) were designated as dependent variables.

To examine experimental results and sources separately, the principal effects of these components, Design-Expert^®^ software (Version 10, Stat-Ease Inc. Minneapolis, MN, USA) was employed, followed by the analysis of variance (ANOVA) to determine each factor’s significance.

### 2.4. Optimization of PRO–UFAs

The desirability function was developed for appropriate formulation selection, aggregating all responses into one variable to forecast the optimum levels of investigated elements [75]. The criteria for selecting the most profitable formulation were achieving the lowest PS value, ZP as an absolute value, and a PDI less than 0.5, in conjunction with the highest EE percentage and Q6h, Table 1.

### 2.5. Preparation of PRO-CTS-UFAs

For the fabrication of PRO–CTS–UFAs, an accurate weight of CTS was dissolved in glacial acetic acid solution at a concentration of 0.03% (*v*/*v*). Then, 2 mL of CTS solution were added dropwise to the previously prepared PRO–UFAs formulation at room temperature while stirring at a magnetically regulated rate. Speeds of 0.2 mL/min and 100 rpm, respectively, were used for the drops and stirring [76]. To verify the PRO–CTS–UFAs’ formation, the previously reported methodologies for PRO–UFAs characterization in terms of EE%, particle size, PDI, ζ potential, and drug release were used.

### 2.6. Transmission Electron Microscopy (TEM)

Transmission electron microscopy was used to investigate the morphological characteristics of the best PRO–UFAs and PRO–CTS–UFAs (JEM-1400, Jeol, Tokyo, Japan). A drop of each dispersion was placed on a copper grid, and the excess was rubbed away with filter paper. The excess aqueous phosphotungstic acid solution (2 percent *w*/*v*, negative staining) was removed similarly. Finally, air-dried samples were examined using TEM at 80 kV [77].

### 2.7. Physical Stability Study

The physical stability of the optimal PRO–UFAs and PRO–CTS–UFAS was analyzed to determine the level of vesicle expansion, drug leakage, and other physical changes. The stability of both formulations was assessed by measuring and comparing the EE percent, PS, and ZP after three months of storage at room temperature. The analyses were conducted in triplicate. The mean and standard deviation were described.

### 2.8. Ex Vivo Permeability Study

#### 2.8.1. Skin Preparation

The dorsal skin of newborn rats weighing 70 ± 20 g was removed after they were killed. The epidermal surface was not harmed by subcutaneous tissues, and adherent fats were removed from the dermal surface. The skin was fixed in the freezer at −20 °C until being used for permeation [78].

#### 2.8.2. Ex Vivo Permeation Study

The permeation of PRO via the skin of rats was compared between optimum UFAs, CTS–UFAs, and PRO solution in phosphate-buffered saline (pH 7.4). Before the investigation, the skin was equilibrated in BPS for 3 h after being defrosted at room temperature. The membrane was placed on a diffusion cell with the SC facing the donor compartment and the dermis facing the receptor compartment. Five cm^2^ of membrane surface area was accessible for diffusion. The donor compartment was loaded with PRO–UFAs, PRO–CTS–UFAs, and PRO solution (3 mg of PRO) in PBS, whereas the receptor compartment was completed to 50 mL of buffer at 32 ± 0.5 °C and swirled at 100 rpm. At predetermined time intervals up to 24 h, 2 mL samples of the receptor fluid were removed, and a new BPS solution was promptly reintroduced to preserve constant volume and sink conditions. The removed samples were then examined at 290 nm using a spectrophotometer.

For each formulation, the cumulative amount of drug permeated per unit area (g/cm^2^) was plotted against time (h). The permeation parameters Q24h in g/cm^2^, permeability coefficient (Kp) in cm/h, and drug flux (Jss) in g/cm^2^ h were calculated for the optimal PRO–UFAs, PRO–CTS–UFAs, and the control PRO solution. In addition, the enhancement index (EI) was calculated using the equation below:

(2) $$I=\frac{\mathrm{Kp}\;\mathrm{of}\;\mathrm{Ufasomes}}{\mathrm{Kp}\;\mathrm{of}\;\mathrm{control}\;\mathrm{solution}}$$

### 2.9. In Vivo Pharmacological Study

#### 2.9.1. Animals

The in vivo experiment was conducted on adult male Wistar rats, whose weight ranged from 180 to 200 g. Animals were provided from Nahda University animal house, Beni-Suef, Egypt. Rats were reserved in an air-conditioned (25 ± 1 °C) pathogen-controlled experimental animal room for two weeks for adaptation before conducting the experiments with free access to standard forage and tap water and libitum.

#### 2.9.2. Experimental Design

Rats were randomized into five weight-matched groups of ten rats for each; a normal control group received vehicles, the sciatic nerve-injured group received cisplatin intraperitoneal for four weeks (2 mg/kg/ twice a week; [21]), and three treatment groups were the standard PRO solution group, PRO–UFA gel group, and PRO–CTS–UFAs gel. Treatments were applied on the rats’ dorsal region to allow systemic drug influence for thirty consecutive days after cisplatin induction at doses (10 mg/kg/day; [48]). The treatment dose was calculated through pilot studies guided by published research.

After 24 h of the last treatment dose was withdrawn, the animals were weighted and sacrificed, and the sciatic nerve removed and preserved in 10% formalin for histoological study and molecular evaluation.

#### 2.9.3. Methods

##### Tissue Sampling

Thiopental sodium (40 mg/kg, ip of 2.5 percent thiopental) was used to anesthetize rats [79]. The rats were then decapitated, and a tight incision on the back was made, exposing the sciatic nerve around the greater sciatic foramen. About four to five sciatic segments were gently isolated and cleaned with ice-cold saline to eliminate blood, rapidly kept in Eppendorf tubes, and fixed at −80 °C until the time biochemical measurement for peripheral myelin 22 real-time PCR assay and malondialdehyde (MDA), glutathione (GSH) and catalase (CAT) ELISA tissue assay. The other sciatic nerve section was preserved in 10% isotonic formalin solution in normal saline until being used in the histopathological study and immunohistochemical assay of apoptosis regulator (BCL-2 associated x), matrix metalloproteinase 9 (MMP-9), and tumor necrosis factor-alpha (TNF).

#### 2.9.4. ELISA of Tissue Biomarkers

MDA, GSH, and CAT levels in sciatic nerve tissue were determined using ELISA test kits and ELISA processing system (SpectraMax Plus-384 Absorbance Microplate Reader, Philadelphia, CT, USA) following the reported sandwich method [80].

#### 2.9.5. Quantitative Peripheral Myelin 22 Real-Time PCR Tissue Biomarkers Assessment

The housekeeping gene, actin, was used as an internal reference in the qRT-PCR technique to calculate fold changes in the target gene from normal rats. The expression of the target gene in normal rats was considered the baseline for calculating fold changes in the target gene from normal rats [81]. All pure RNA from a homogenized, isolated sciatic nerve, homogenized in lysis buffer, per the manufacturer’s instructions, was analyzed. The purification column was initially loaded, then the desired amount of pure RNA was extracted and tested for purity using a UV-Vis spectrophotometer Q5000 (Quawell Technology, Inc., Sunnyvale, CA, USA) at OD260/280 nm and the nanodrop method. A reverse transcriptase kit converted RNA to its corresponding DNA (cDNA) following the manufacturer’s instructions. Expressed genes were quantified using 2X Maxima SYBR Green/ROX qRT-PCR Master Mix to amplify cDNA according to the manufacturer’s procedure and specific gene-specific primers, as in Table 3.

#### 2.9.6. Histopathological Study

The sciatic nerve was fixed in a 10% isotonic formalin solution for histological evaluation. After ensuring tissue stiffening, samples were treated using the Bancroft and Steven 1983 paraffin embedding procedure, through dehydrating in graded ethyl alcohol (50, 70, 95, and 100%) for 2 h each and cleared in two changes of xylene after the fixation process. Before blocking, samples were primarily embedded in paraffin wax three times for two hours each time. Sections of 5 µm tissue were cut using the Leica microtome. Sections stained with Hematoxylin and Eosin were examined under a light microscope with the aid of a pathologist [82].

#### 2.9.7. Immunohistochemical Assay

The BCL-2, MMP, and TNF α tissue assays were performed according to the previously described assay technique [83,84]. In brief, paraffin-embedded tissues of 5-μm thickening were dehydrated in xylene, followed by graduated ethanol concentrations. Slides were blocked for 2 h with 5% bovine serum albumin (BSA) in Tris-buffered saline for immunostaining (TBS). The immune-staining with primary antibodies against BCL-2, MMP, and TNF α were incubated overnight at 4 °C and then washed with TBS before adding secondary antibodies. Then, 0.02% diaminobenzidine H_2_O_2_ was added for 10 min. A histopathologist helped with the hematoxylin counterstaining before the slides were inspected under a light microscope.

#### 2.9.8. Statistical Analysis

All data were expressed as means of 6–10 values ± standard error (SEM). Statistical analyses were performed using a one-way analysis of variance test (ANOVA) followed by the Tukey–Kramer multiple comparisons test using a statistical package for social sciences software (SPSS; version 19.0), a computer program offered by (SPSS Inc., Chicago, IL, USA), where the value of *p* < 0.05 was considered statistically significant.

## 3. Results

### 3.1. Analysis of Factorial Design

Identifying the variables that may affect the properties of a newly developed drug delivery system is essential. In this regard, factorial designs are advantageous because they can simultaneously examine the effect of multiple factors on the parameters of the drug delivery system [85]. In the present investigation, the ranges of the independent variables were assessed using preliminary tests (data not displayed) that were used to select the variables and their levels. The chosen model was two-factor interaction (2 FI). For each investigated response, the predicted R^2^ values correlated reasonably well with the adjusted R^2^ values (Table 4). All responses exhibited adequate precision with a ratio greater than 4 (the desired value), ensuring that the model could be utilized to navigate the design space [86].

### 3.2. PRO–UFAs Characterization

#### 3.2.1. Effect of Formulation Variables on EE%

**Figure 1:**
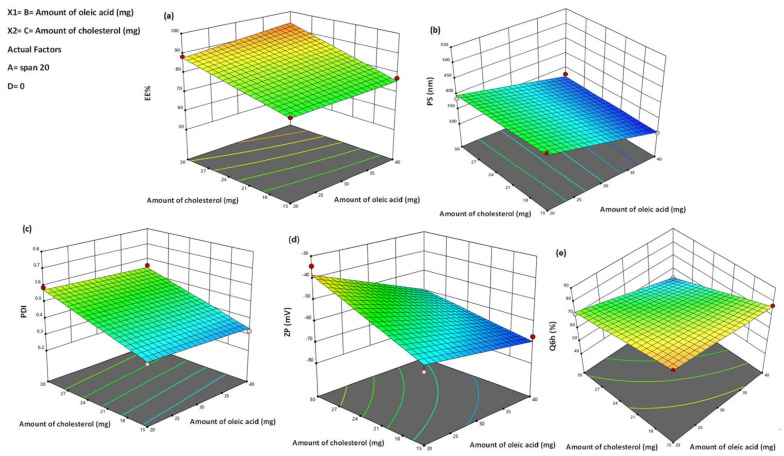
Response surface plot for the effect of oleic acid amount (B), cholesterol amount (C) at the middle levels of the 1st and 4th variables (Span type and sonication time) on (**a**) EE%, (**b**) particle size, (**c**) PDI, (**d**) zeta potential, and (**e**) Q6h of the developed UFAs’ dispersions.

The higher drug capture within the vesicle’s assembly is required to provide an acceptable drug amount [87]. In this research, the proportion of PRO engaged by UFA formulations ranged from 53.64 ± 1.04% to 97.52 ± 4.05% (Table 2). The combined influence of the two independent variables (cholesterol and oleic acid amounts) on the EE percent of PRO–UFAs at the low and intermediate levels of the first and fourth variables (span type and sonication time) is graphically depicted in Figure 1a as 3D surface plots.

Analysis of variance revealed that the span type (A) significantly influenced the EE percentage (*p* = 0.0014). The EE percent values of Span 60-containing UFAs formulations were higher than those of Span 20, which might be attributable to the solid nature, hydrophobicity, and higher phase transition temperature of Span 60, which allowed for a higher degree of drug encapsulation [88]. It was reported that the encapsulation efficiency increased as the transition temperature of the surfactant increased [89]. The increase in alkyl chain length, Span 60 (C18) > Span 20 (C12), has led to a rise in the EE percentage, as previously reported [90,91,92]. In addition, the alkyl chain length affected the hydrophilic-lipophilic balance (HLB) value of the surfactant, which directly influenced the drug EE percent [93]. The lower the HLB of the surfactant, the greater the drug EE percent and stability [94], as in the case of niosomes prepared using Span 60, HLB = 4.7 compared to Span 20, HLB = 8.6.

There was a positive effect of oleic acid on PRPO EE%. The presence of oleic acid in the lipid bilayer could modulate the crystallization array due to its unsaturation and lipid trait conferring comparative imperfection in the bilayer chains, thus maintaining the laden drug with avoidance of its expulsion. [95]. In addition, it was determined that the presence of oleic acid in the vesicles’ nano-cargo could provide proper space to host the drug particles in the amorphous network of the system, resulting in an increased EE percent [72]. This result was consistent with that of Gabr et al. [96], who stated that the EE percent of rosuvastatin increased with oleic acid addition to the lipid domain in the formulation of hexagonal liquid crystalline nanoformulation, thereby allowing for a greater drug entrapment.

Cholesterol level was found to impact EE percentage (*p* = 0.0003) significantly. An increase in cholesterol level led to a rise in the entrapped drug. Our findings are consistent with those of Khalilet al. and El-Nabarawi et al., who asserted that the EE percentage of bilosomes increased as cholesterol levels increased [97,98]. Generally, cholesterol makes lipid bilayer membranes more hydrophobic and rigid, resulting in highly organized vesicles with excellent membrane stability, reduced drug penetration, and, consequently, higher drug retention [78].

Matloub et al. [99] proposed an alternative explanation for the increase in EE percentage of bilosomes when the amount of cholesterol is increased. Cholesterol could prevent the gel-to-liquid phase transition of the surfactant bilayer, enhancing the vesicle membrane’s microviscosity. This stabilizes the hydrophobic bilayer, stops drug leakage, and increases the vesicle’s EE percentage [99].

It was also evident that sonication time significantly impacted the EE percent values of PRO–UFAs. The exposure of UFAs to sonication for 15 min significantly decreased the percentage of PRO EE (*p* = 0.001). This could be attributed to vesicle disruption and re-agglomeration, with concomitant evasion of a large quantity of the drug to the external aqueous medium containing cholesterol and, thus, being kept in the aqueous milieu via micellar solubilisation rather than being encapsulated within UFAs. This result is consistent with El Menshawe et al. [100], who reported the reduction in EE percentage upon the spanlastics formulation for fluvastatin delivery with an increase in sonication time.

#### 3.2.2. Effect of Formulation Variables on PS

PS is an essential parameter in developing a transdermal vesicular delivery system because it influences the penetration of vesicles over the skin. Multiple trails have assured that small-sized vesicles penetrate the skin deeper than larger ones [101]. Consequently, one of our goals was to fabricate vesicles with smaller particle sizes to ensure deeper penetration within the epidermal layer [102]. Table 2 showed that the prepared UFAs varied between 302.78 ± 18.52 nm and 510.44 ± 20.02 nm, placing them within the nano-scale range. The effects of span type (A), oleic acid amount (B), cholesterol amount (C), and sonication time (D) on the PS of UFAs were graphically depicted in Figure 1b as 3D surface plots. According to the studied design, the elements that contributed to boosting the EE%, namely span type (A) and oleic acid amount (B), had also greatly enhanced the vesicle size. There is a direct correlation between the vesicle size and drug entrapment as the bilayer’s distance rises due to the inclusion of the drug in the hydrophilic zone of the vesicles [103]. The average size of UFAs based on Span 60 was greater than that of vesicles based on Span 20. Presumably, the larger the vesicle size produced, the longer the alkyl chains and the lower the HLB. Thus, span 20-based UFAs (C12 and HLB = 8.6) were smaller than Span 60-based UFAs (C18 and HLB = 4.7). These outcomes are comparable to those reported [104,105].

In addition, the increase in cholesterol (C) leads to an escalation in the mean PS. Liposome vesicles with a high cholesterol level are less likely to pack tightly together, showing a more even distribution of the aqueous phase and an increase in PS [106]. Furthermore, a higher drug load within the UFAs was linked to the elevated cholesterol levels, which may have also contributed to the enlarged vesicles.

Figure 1b demonstrated that an increase in oleic acid amount (B) was associated with a significant enhancement in the particle size of the formulated PRO–UFAs (*p* = 0.033). This relative increase in size might be attributable to the increased viscosity caused by the increase in oleic acid amount. When the EE percent values were considered, these results were not surprising, as higher oleic acid content was associated with a greater amount of PRO encased within the vesicles, and thus a larger vesicle size was formed. Pinilla et al. [107] reported that adding oleic acid to freeze-dried liposomes containing natural antimicrobials increased nanovesicle size and potential. Kelidari et al. [108] realized a reduction in particle size alongside an increase in oleic acid concentration during the preparation of spironolactone nanoparticles. These contradictory results might be explained by the differences in the nature and affinity of lipid in the various drugs utilized. After sonicating PRO–UFAs for 15 min, the vesicles’ size decreased significantly, as expected. This could be attributed to vesicle exposure to ultrasonic waves, which led to the dispersion of UFAs into smaller sizes [109]. Such findings are supported by previous literature [110,111].

#### 3.2.3. Effect of Formulation Variables on PDI

A PDI value ranged from 0 to 1, with 0 denoting extremely monodispersed particles and 1 denoting highly polydispersed vesicles [112]. All UFA formulations had PDI values between 0.28 ± 0.023 and 0.71 ± 0.016, indicating an excellent homogeneity and narrow size distribution (Table 2). The influences of span type (A), oleic acid amount (B), cholesterol amount (C), and sonication time (D) on the PDI of UFAs were graphically represented in Figure 1c as 3D surface plots. An analysis of variance revealed significant effects of span type (A) (*p* = 0.0001), cholesterol amount (C) (*p* = 0.0006), and sonication time (D) (*p* = 0.0012) on PDI, whereas oleic acid amount (B) had no effect. This suggested PDI of UFA dispersions was impacted by the same parameters that affected its PS. Regarding the span type (A), it was evident that UFAs formulated with Span 20 had the lowest PDI values, most likely due to smaller PS and lower EE percentage compared to UFAs prepared with Span 60. It was indicated that there was a direct correlation between cholesterol quantity (C) and PDI. An increase in cholesterol amount (C) resulted in an increase in drug entrapment within the vesicles, leading an increase in vesicle heterogeneity. In addition, Aithal et al. highlighted the increase in PDI of liposomes with the increase in the molar ratio of cholesterol [113].

Furthermore, sonication time (D) had a statistically significant negative impact on UFAs PDI, resulting in a decrease in particle size and PDI when samples were subjected to prolonged sonication in the final stages of UFAs formation. This was consistent with the results of de Freitas et al., who found that as sonication time increased during the preparation of small unilamellar vesicles, the size and PDI values decreased [114]. Chen et al. [115] claimed that sonication time was the most important factor influencing particle size and PDI. There was an inverse relationship between sonication time, particle size, and PDI during the preparation of niosomes uploaded with diacerein [111]. In contrast, the amount of oleic acid (B) had no significant effect on the PDI of UFAs.

#### 3.2.4. Effect Formulation Variables on Zeta Potential (ZP)

The ZP has a vital impact on the storage stability of particle dispersions. When the ZP value of a system is approximately ±30 mV, it is generally regarded as stable because of the electric repulsion between the particles [116]. This confirms that the vesicles have adequate charges to prevent aggregation due to electric repulsion. In this investigation, the charge properties on the surface of the prepared UFA dispersions were examined, and the outcomes revealed negative charges with ZP values on their surfaces fluctuating from –31.15 ± 1.13 mV to −71.12 ± 1.50 mV (Table 2). Since formulations in our investigation had negative ZP, ZP values will be presented in absolute terms to avoid misunderstanding. The high zeta potential value is attributed to surfactant lipophilicity and the presence of oleic acid, which increases zeta value. Abd-Elal et al. [117], in a similar paper, evaluated zolmitriptan in trans-nasal novasome formulations. They reported that zeta potential values ranged from 51.57 ± 2.02 to 68.10 ± 10.18 mV [117]. Figure 1d demonstrated that the investigated independent variables significantly affected on the ZP among the various dispersions (*p* = 0.05).

The results indicated that the type of span (A) significantly affected the ZP of PRO–UFA dispersions (*p* = 0.0001). The Span 20-prepared UFAs exhibited the highest absolute ZP, likely due to the lipophilicity of the surfactant-forming vesicles. Due to the decrease in the surface free energy, decreasing the surfactant’s lipophilicity increased ZP values. Span 20 (HLB = 8.6) was less lipophilic than Span 60 (HLB = 4.7), so its ZP values were greater than those of Span 60 [118]. In addition, increasing the amount of oleic acid led to significantly increased ZP values (*p* = 0.038). Manca et al. obtained comparable outcomes on the formulation of rifampicin liposomes [119].

The cholesterol used in UFAs formation had the ability to fill the molecular pores formed by the span, thereby increasing the rigidity of the bilayer membrane, which had a negative effect on the ZP percentage [120]. Thus, adding cholesterol decreased the system’s zeta potential and weakened the repulsion between vesicles, resulting in aggregate formation. However, the sonication time (D) did not significantly influence the ZP of the UFAs (*p* = 0.143).

#### 3.2.5. Effect of Formulation Variables in In Vitro Drug Release Studies

The profiles of PRO release from the produced UFAs dispersions and its solution in Sorensen’s phosphate buffer at pH 5.5 were depicted and 95.76 ± 5.12% of PRO was released from the aqueous solution within one hour, indicating that the inspected dialysis membrane did not prevent drug release. The percentage of PRO released from UFAs after six hours varied between 49.61 ± 0.98 percent and 87.75 ± 3.98 percent, as shown in Table 2. The release profiles of PRO among various UFAs were successful in delaying the PRO solution’s release compared to other UFAs profiles. All UFAs exhibited biphasic release of PRO, with a relatively rapid initial phase followed by a slower phase. The observed burst effect could be attributable to the fast partitioning of the hydrophilic, surface-adsorbed PRO into the releasing medium [104]. This suggested that PRO–UFAs should exhibit a rapid action and prolonged drug delivery due to their initial rapid release and slower phases.

The investigated independent variables significantly affected the Q6h for each dispersion (*p* < 0.05) (Figure 1e). Regarding span type, the Q6h values of UFAs composed of Span 20 were significantly greater than those of UFAs composed of Span 60 (*p* = 0.0007). As previously stated, the rapid release of Span 20-based UFAs could be due to Span 20′s less hydrophobic character compared to Span 60, which promoted the diffusion of the drug to the release medium. In addition, the lower transition temperature of Span 20 might be a contributing factor to the observed increased release. Span 60 has a transition temperature of 53 °C, compared to 16 °C for Span 20 [121]. As the release investigation was conducted at 32 ± 0.5 °C, the reduced release rates of Span 60-composed vesicles might be attributed to their higher phase transition temperature, which effectively placed them in a highly ordered gel state. Elsherif et al. [109] prepared terbinafine hydrochloride–spanlastics, which corresponded to these findings.

Notably, oleic acid-decorated vesicles containing 20 mg oleic acid produced significantly higher release rates than those containing 40 mg oleic acid (*p* = 0.0006). This might be attributed to the increased formation of oleic acid micelles, which are believed to have a slower effect on drug release than vesicles [122]. In addition, reducing the amount of oleic acid in UFA dispersions would produce smaller vesicles with a greater surface area exposed to the release environment, thereby enhancing PRO release. Such outcomes are supported in literature [72].

The cholesterol amount (C) had a significant negative impact on the percentage of PRO release (*p* ˂ 0.0001). Our findings agreed with those of Khalil et al. [98] and Ruckmani and Sankar [123], who observed a significant reduction in the drug release with higher cholesterol amount. The incorporation of cholesterol during the fabrication of UFAs increased lipid packing while decreasing bilayer fluidity and deformability, increasing bilayer rigidity, decreasing drug leakage, and increasing vesicle stability [99,124]. Khelashvili et al. explained the high mechanical stiffness of vesicle membranes due to cholesterol incorporation [125]. They proposed that the cholesterol particle’s structure, four fused cyclohexane rings attached to a hydroxyl group and a hydrophobic tail, allows cholesterol to be contained within the bilayer, where the steroid ring would align parallel to the membrane phospholipid’s hydrocarbon chains. A hydrogen bonding would occur between the hydroxyl group and the phospholipid polar head groups. The rigid steroid ring would interact with the hydrocarbon chains, contrasting the splay mode of deformation between pairs of lipids and cholesterol. This clarification is consistent with that of Ayee and Levitan [126].

The time of sonication had a significant positive effect on the Q6h of vesicles containing UFAs, according to statistical analysis of the release data (*p* = 0.0011). This link between sonication time and Q6h could be attributed to particle size, as the proportion of the drug dispersed in the aqueous medium at a particular time is inversely proportional to the size of the vesicles. Thus, the smaller vesicles that were produced could decrease the diffusional distance of the drug, thereby increasing drug release rates [127].

A mathematical analysis of PRO release data revealed that the Higuchi kinetics release model governed drug release from most formulated dispersions, indicating a diffusion-controlled mechanism. According to some studies, drug-based vesicular systems that follow Higuchi’s square root model facilitate controlled drug release [70,72].

To clarify PRO release kinetics, the Korsmeyer–Peppas model was used, to lighten other drug release mechanisms. In the Korsmeyer-–Peppas equation, the n values for Fickian (diffusional), zero-order release kinetics, and non-Fickian (anomalous) release are 0.5, 1, and 0.5 ˂ *n* ˂ 1, respectively. In our investigation, the n values for the various dispersions ranged from 0.55 to 0.99, indicating a non-Fickian drug diffusion and atypical drug release pattern in which drug diffusion and lipid bilayer distention might be combined [128].

### 3.3. Selection of the Optimized Formulation

Utilizing Design-Expert software, the optimal formulation was chosen based on a full factorial design. The formulation prepared using the combination of Span 20 and oleic acid (40 mg *w*/*w*) in the presence of 22.52 mg *w*/*w* cholesterol without sonication met the requirements for an optimal formulation (achieving maximum values of EE percent and Q6h and minimum values of PS, ZP (absolute value), and PDI less than 0.5. As depicted in Table 5, this formulation exhibited EE percent values of 82.72 ± 2.33 percent, PS values of 317.22 ± 6.43 nm, PDI values of 0.441 + 0.02, ZP values of −62.06 ± 0.07 mV, and Q6h values of 70.95 ± 8.14 percent. Therefore, it was chosen as the optimal performing formulation for further research.

### 3.4. Formulation and Characterization of PRO–CTS–UFAs

Based on the most effective PRO–UFAs, PRO–CTS–UFAs were created. As shown in Table 5, the influence of CTS nanoparticles on dependent variables such as EE percent (Y1) and particle size (Y2) and potential was investigated. The optimal PRO–UFAs and PRO–CTS–UFAs had a particle size of 405.22 ± 6.43 and 424.12 ± 4.9 nm, respectively. This increase in PS could validate the coating procedure. EE values for PRO–UFAs and PRO–CTS–UFAs were 82.72 ± 2.33 percent and 85.32 ± 2.65 percent, respectively. In addition, the zeta potential of freshly prepared optimal PRO–CTS–UFAs of 65.24 ± 0.10 mV represented quality dispersion. The coating of UFAs by CTS altered negative zeta potential values to positive values. In fact, the greatness of the zeta potential is an excellent indicator of a colloidal system’s overall stability [129], while Q6h was performed and found to be 70.95 ± 8.14% and 64.03 ± 1.9% for optimal PRO–UFAs and PRO–CTS–UFAs, respectively. The decrease in Q6h could be attributed to the increase in PS.

### 3.5. Transmission Electron Microscopy (TEM)

**Figure 2:**
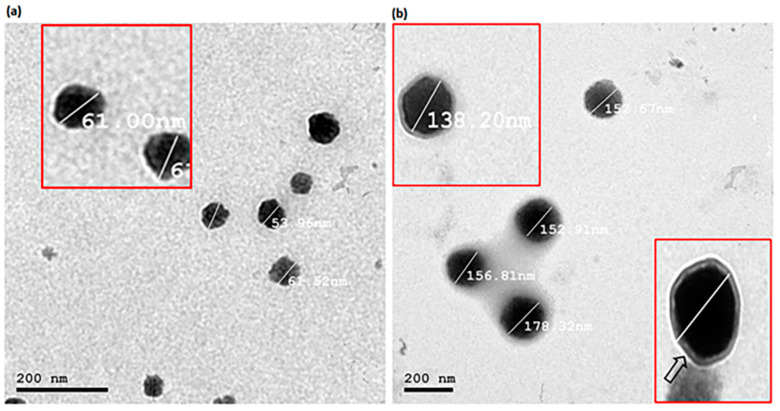
Transmission electron photomicrographs of (**a**) PRO–UFAs and (**b**) PRO–CTS–UFAs.

TEM analysis is a useful method for determining the nanovesicles’ shape, lamellarity, and size [130]. Figure 2 depicts TEM micrographs of the optimized PRO–UFAs and PRO–CTS–UFAs. The hypothesized vesicles were nanostructured, spherical, unilamellar, non-agglomerating, and uniformly sized. The photomicrograph of PRO–CTS–UFAs, as shown in Figure 2b, revealed a minor increase in particle size, elucidating the adsorption of the CTS coat, which appeared as a very thin layer encircling the UFAs’ shell. Due to the different analysis principles involved in each technique, the size obtained by transmission electron microscopy (TEM) is smaller than that obtained by dynamic light scattering using a Zetasizer NanoZS (Malvern Instrument). The resultant size distribution of dynamic light scattering (DLS) is the average hydrodynamic size of the nanoparticles and is frequently influenced by the presence of large particles, dust, or aggregates [131]. The nanoparticles measured by DLS techniques, in particular, are in solution surrounded by nonmoving layers of the used medium, which increases their measured diameter. However, microscopic investigation by TEM is mostly based on nanoparticle tracking analysis (NTA), and observations are typically conducted following nanoparticle droplet air-drying on the TEM grid as a standard technique. NTA is a technology that uses numbers to track single nanoparticles (single-particle tracking) [131]. The latter can therefore provide an accurate number-based average dimension with minimal bias for artifact-free samples [132]. Consequently, DLS analysis will yield a larger size than TEM analysis.

### 3.6. Physical Stability Study

**Figure 3:**
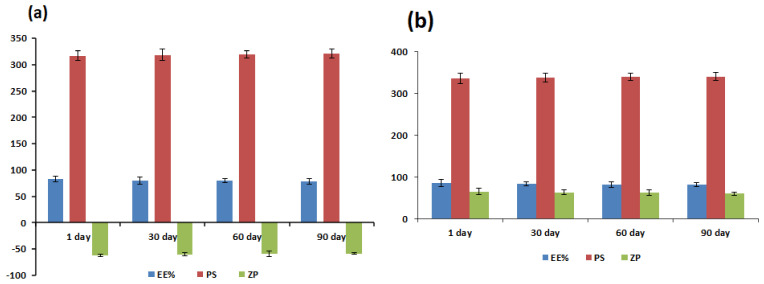
Effect of storage on EE%, particle size, and ζ potential of (**a**) PRO–UFAs and (**b**) PRO–CTS–UFAs.

The EE percentage, vesicle size, and zeta potential of the optimized PRO–UFAs and PRO–CTS–UFAs were assessed as the primary storage stability parameters after 30, 60, and 90 days. During storage, both aggregation and abnormality were not observed. As depicted in Figure 3, the PRO EE percentage, vesicle size, and potential did not change significantly during the 90 days storage (*p* > 0.05), indicating that the nanoformulations were kinetically stable. The apparent increased stability highlights the significance of the oleic acid/span/cholesterol combination. To keep the ufasomal membrane stable, cholesterol prevents the polar head groups of SPC in the bilayer from interfering with one other’s electrical shells and increases the distance between the phospholipid chains [133]. In addition, this high stability could be attributed to the custom-made nanovesicles’ small particle size and narrow size distribution. Additionally, PRO–UFAs and PRO–CTS–UFAs exhibited a high potential (>25 mV), which may contribute to colloidal stability and prevent vesicle aggregation [134]. As shown in Figure 3b, CTS’s protective layer-covered vesicles contributed to overall stability [134]. Our findings indicated that the amount of CTS necessary to coat particles with opposite charges in the optimized formulation was sufficient to produce a stable dispersion without a separation phase.

### 3.7. Ex Vivo Skin Permeation Study

**Figure 4:**
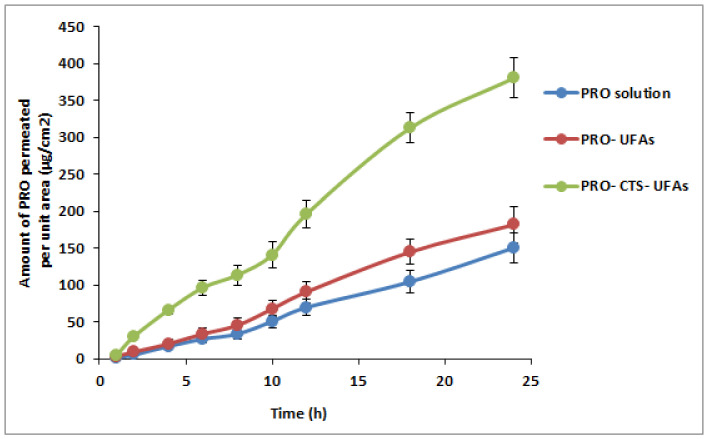
Ex vivo permeation study of PRO–CTS–UFAs compared to PRO solution and PRO–UFAs.

Ex vivo permeation studies shed light on the in vivo effectiveness of a transdermal medication delivery method. The permeability of PRO from UFAs and PRO-CTS-UFAs via excised skin was investigated to compare its permeation profile to that of PRO solution. The cumulative amount of PRO penetrated per unit area through chosen UFAs and CTS–UFAs relative to PRO solution as a function of time was depicted in Figure 4. As shown in Table 6, CTS–UFAs had Q24 values of 380.05 ± 13.4 (g/cm^2^), Kp values of 0.0169 ± 0.0007 (cm/hr), lag times of 50.63 ± 2.23 min, and steady-state flux values of 16.98 ± 0.12 (g/cm^2^/h) compared to 181.61 ± 10.5 (g/cm^2^) and Kp values of 0.00812. In reality, oleic acid could potentially disrupt the skin barrier. According to other studies [135,136], oleic acid can disrupt the epidermal barrier by dissolving the stratum corneum’s lipid chain. This discovery is consistent with previous ones. Rowat and colleagues discovered that oleic acid can cause phase separation in a simulated stratum corneum membrane containing bovine brain ceramide, cholesterol, and palmitic acid, which changes the structure and permeability of the stratum corneum [137]. Since oleic acid increases skin permeation by stimulating epidermal lipid bilayer fluidization and corneocyte shrinkage via keratin condensation [138], resulting in the enlargement of aqueous pores for transdermal drug delivery [138], oleic acid-containing vesicles are expected to enable hydrophilic drug transportation through the skin. In addition, the surface charge of the PRO–CTS–UFAs was the essential aspect in deciding how the CTS may improve skin drug delivery. [139,140]. CTS coating conferred a positive surface charge to UFAs, which provided a crucial function in interacting with the SC’s negative charge to enhance the diffusion of the drug. The potential for positive polymer CTS to disrupt the tight connections of negative charges in the skin, accelerating the distribution of PRO–CTS–UFAs, is another hypothesized mechanism [141]. Moreover, the bio-adhesion force of CTS caused the vesicle to remain in contact with the skin for a longer period, leading to higher skin diffusion and penetration [92].

### 3.8. In Vivo Pharmacological Study

#### 3.8.1. Biochemical Measurement

##### Catalase Activity

**Figure 5:**
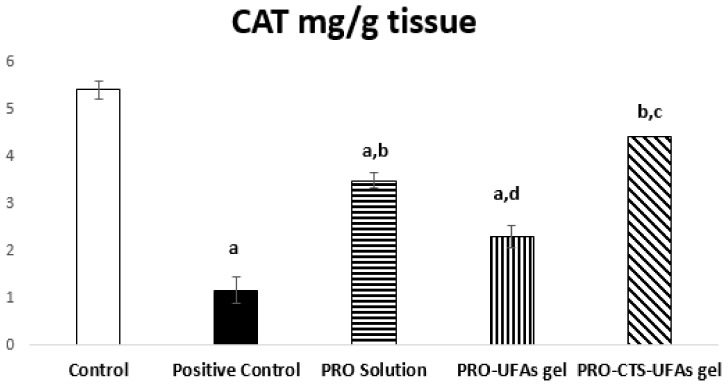
Effect of thirty days’ treatment with PRO solution, PRO–UFAs gel, and PRO–CTS–UFAs gel on sciatic nerve catalase activity in cisplatin-induced neuropathy. Values are mean ±  SD (*n* = 8). Data were analyzed by one-way ANOVA followed by Post Hoc Tukey for multiple comparisons. ANOVA; ^a^ Significant difference in comparison with the control group. ^b^ Significant difference in comparison with cisplatin positive control group ^c^ Significant difference in comparison with PRO–UFAs gel and ^d^ Significant difference in comparison with PRO–CTS–UFAs gel at (*p* < 0.05).

In the current study, we aimed to assess a new preparation of PRO–CTS–UFAs gel against sciatic nerve neurological disorder induced via subjecting male albino rats to cisplatin, compared to PRO–UFAs gel and PRO solution. Since it keeps drug concentration within the therapeutic window for an extended period of time, the transdermal formulation can ensure that medication levels do not fall below the minimum effective concentration or rise above the maximum effective concentration [142]. Thus, our data presented that normal catalase levels is 5.40 ± 0.14 mg/g tissue. In contrast, cisplatin induction was significantly decreased to 1.16 ± 0.19 mg/g tissue (21.48%) compared to normal control rats, where treatments with PRO solution and PRO–UFAs gel improved tissue levels to 3.49 ± 0.28 mg/g tissue (300.86%) and 2.31 ± 0.17 mg/g tissue (199.14%), respectively, compared to the positive control. In rats treated with PRO–CTS–UFA gel, their CAT tissue levels were approximately restored to normal levels of 4.42 ± 0.23 mg/g tissue (381.03%) compared to the cisplatin group, Figure 5.

Khodaei et al. [143] demonstrated that mice multiple sclerosis model induction significantly affected catalase protein levels in both the sciatic nerve and spinal cord. Additionally, a cisplatin-induced neuropathy model affected catalase levels protected by co-administration of melatonin, revealing its potent antioxidant activity [144]. Recently, the role of propranolol in tumor suppression oxidative stress in neural macrophages has been reported by targeting β adrenergic receptors [145]. Its neuroprotective effect in several models of transient focal stroke was attributed to its antioxidant and free radical scavenger properties [146,147]. Propranolol, a potent membrane anti-peroxidative, exhibited cardio-protection against the ischemia/reperfusion rat model restoring catalase content [148,149]. Moreover, propranolol administration reduces post-traumatic brain injury mobilization and microvascular permeability in the murine penumbral neuro vasculature, cerebral edema, and brain oxidative stress [149]. Interestingly, beta-blockade was reported to have antioxidant potentials in different models, owing to the regulation of mitochondrial poly-ADP-Ribose polymerase/cAMP/protein kinase A axis [48,150]. Finally, propranolol could act as a transdermal PNI treatment agent based on its antioxidant effect, evident in the current study, from significant corrections in tissue levels of CAT in cisplatin PNI rats receiving propranolol Figure 5.

##### Oxidative Markers

**Figure 6:**
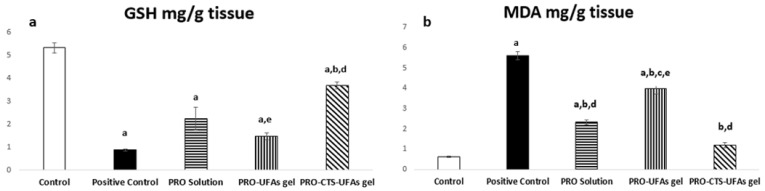
Effect of thirty days of treatment with PRO solution, PRO–UFAs gel, and PRO–CTS–UFAs gel on sciatic nerve GSH (**a**) and MDA (**b**) against cisplatin-induced neuropathy. Values are mean ± SD (*n* = 8). Data were analyzed by one-way ANOVA followed by Post Hoc Tukey for multiple comparisons. ANOVA; ^a^ Significant difference in comparison with the control group. ^b^ Significant difference in comparison with cisplatin positive control group. ^c^ Significant difference in comparison with PRO solution, ^d^ significant difference in comparison with PRO–UFAs gel, and ^e^ significant difference in comparison with PRO–CTS–UFAs gel at (*p* < 0.05.).

GSH is a natural endogenous antioxidant produced by the liver as a peroxidase scavenger [151]. Consequently, it plays an essential role in treating cisplatin-induced sciatic nerve impairment. The antioxidant effect of PRO-CTS–UFAs gel was refected by regulating both GSH and MDA. There was a significant increase in GSH by 420.09% and mitigation of MDA by 21.15% at (*p* < 0.05) compared to the cisplatin positive control group, while restored MDA levels to normal with a significant GSH difference at (*p* < 0.05) compared to the normal control group (Figure 6a,b). PRO solution and PRO–UFAs gel had improved tissue levels of GSH by 258.62% and 168.97% and MDA by 41.46% and 70.99%, respectively, compared to the cisplatin positive control group at (*p* < 0.05), as shown in Figure 6.

Previous research has found that propranolol reduces oxidative stress and inflammation [152,153]. Propranolol has been reported as a potent antioxidant due to its ability to suppress superoxide anions that have beneficial effects on endothelial dysfunction treatment [154]. β-adrenergic antagonists are extensively expressed in peripheral neurons and play an essential role in controlling chronic pain [155]. Previous data revealed that β1-, β2-, and β3 receptors in the osteosarcoma mouse model contribute to the presence of pain, but by administration of beta blockers, the pain was diminished [156]. All these studies confirmed our results on propranolol neurological role.

Furthermore, Abdel Salam et al. [157] revealed that concurrent administration of the propranolol was associated with reduced liver injury, involving decreased hepatic oxidative stress. Abdel-Wahab et al. [158] demonstrated the cardioprotective effects of propranolol on clozapine-induced myocarditis by inhibiting oxidative stress, inflammation, and reducing cell apoptosis. In agreement, the antioxidant effect of non-selective adrenergic antagonist carvedilol was reported in an animal model of brain injury [159]. Additionally, it was reported that β-blockers could protect against experimentally induced hepatotoxicity [160] and nephrotoxicity [161], owing to their antioxidant potential. Sherif et al. [162] recommended the addition of propranolol in acute theophylline toxicity, proving the oxidative stress mechanism’s role in theophylline toxicity.

##### Gene Expression of Peripheral Myelin 22 by the Real-Time PCR

**Figure 7:**
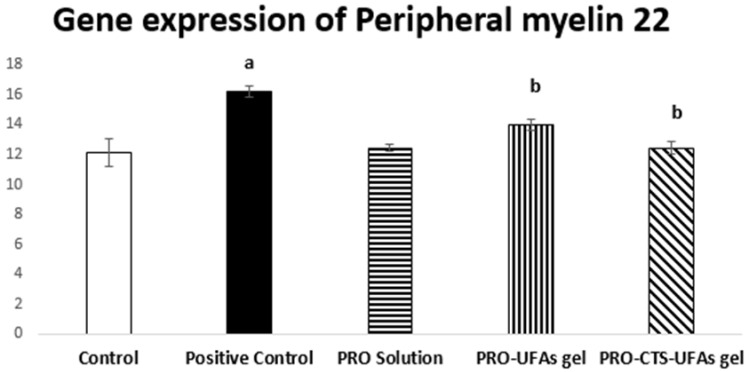
Effect of thirty days’ treatment with PRO solution, PRO–UFAs gel, and PRO–CTS–UFAs gel on sciatic nerve peripheral myelin 22 gene expression against cisplatin-induced neuropathy. Values are mean ± SD (*n* = 8). Data were analyzed by one-way ANOVA followed by Post Hoc Tukey for multiple comparisons. ANOVA; ^a^ Significant difference in comparison with the control group. ^b^ Significant difference in comparison with cisplatin positive control group at (*p* < 0.05).

The human nervous system consists of a unique structure of myelin sheaths that contributes to electrical nerve signals transmission and participates in many other vital physiological effects [163]. Therefore, nerve impulse conduction in the case of myelinated nerve fibers is faster than with the unmyelinated ones [164]. Formerly, if they are injured, it will take a long time to repair and remyelinate, a typical pathological manifestation of peripheral neuronal damage [165]. On that basis, we hypothesized that cisplatin-induced sciatic nerve injury leads to peripheral myelin 22 damage due to neuronal disorder. Our results indicated that the mean value of peripheral myelin 22 of the rats subjected to cisplatin was 16.19 ± 0.36, significantly higher than that of the control group by 133.51%. PRO–UFAs gel treated group showed significantly lower peripheral myeline 22 gene expression group by 86.41% compared to cisplatin positive control group, while the PRO–CTS–UFAs gel and PRO solution treated group restored peripheral myelin 22 levels almost to normal compared to the cisplatin positive control group, Figure 7.

Additionally, it was reported that the highly expressed adrenaline and noradrenaline, as in multiple sclerosis, induced the proliferation of the specific neoantigens in the draining lymph nodes [166]. Furthermore, the experimentally induced stroke mice model revealed the trigger of sympathetic stimulation-induced immunodeficiency as a defense mechanism for inflammation and infection-induced injury [167]. Indeed, the sympathetic nervous system developed a key regulatory role in modulating the immune system either in steady-state or in inflammation and tissue damage [168]. All these previous works confirmed that we could proceed with immunomodulatory, antioxidant, anti-inflammatory, and anti-apoptotic effects by inhibiting adrenergic activity.

#### 3.8.2. Histopathology

##### H&E Staining

**Figure 8:**
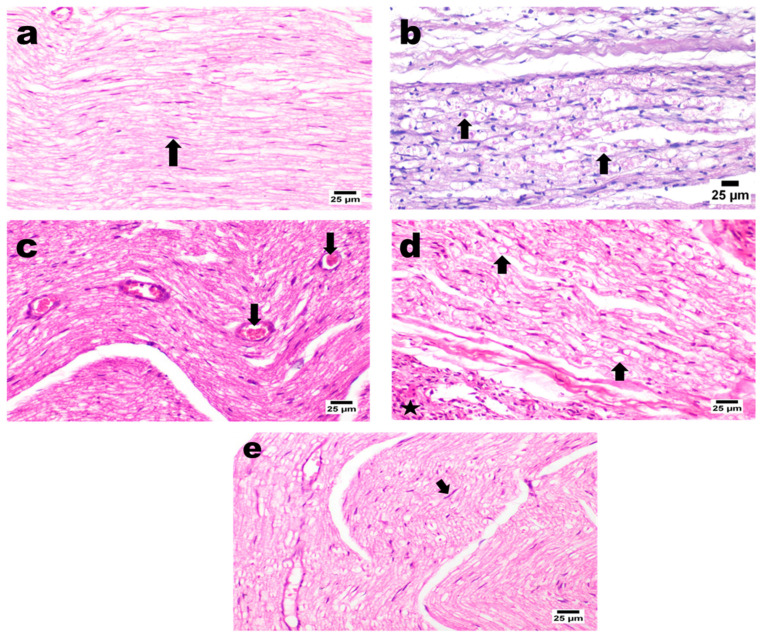
Photomicrographs of sciatic nerve of 30-days daily dorsal application administration of PRO solution, PRO–UFAs gel, and PRO–CTS–UFAs gel against cisplatin-induced sciatic nerve injury (H&E; 25x); Top left (**a**) A normal control rat section showing normal histological structure of myelinated nerve fibers (black arrow); Top middle (**b**) An cisplatin positive control rat section showing demyelination of nerve fibers with a Wallerian degeneration coupled with significant presence of abundant edema with inflammatory cells infiltration in the surrounding tissue (black arrows); Top right (**c**) PRO solution group revealed numerous congested blood capillaries coupled with mild inflammation in the nerve sheath (black arrows); Bottom left (**d**) PRO–UFAs gel section represents demyelination and vacuolated nerve fibers (black arrows), with a numerous mononuclear inflammatory cells infiltration in the perineuronal tissue (black star); Bottom middle (**e**) PRO–CTS–UFAs gel investigation apparently with normal myelinated nerve fibers in several examined sections coupled with few mildly dilated of endoneurial blood vessels associated with mild inflammation in the perineuronal tissue (black arrow).

Sciatic nerve sections obtained from normal control rats showed the normal histological structure of the sciatic nerve, expressed as closely packed nerve fibers with occasional endoneurial blood vessels and each individual nerve fiber and a central axon surrounded by a sheath of myelin. Rats subjected to intraperitoneal injection of cisplatin reveal marked nerve fibers’ demyelination associated with Wallerian degeneration and vacuolation of nerve fibers, along with the dispersion of nerve fibers with excessive edema and observed perineuritis in some areas characterized by numerous mononuclear inflammatory cells’ infiltration (Figure 8).

On the other hand, PRO solution-treated rats showed mild to moderate perineuritis with mild congested blood capillaries and severe myositis. This is characterized by numerous mononuclear inflammatory cell infiltrations associated with the necrosis of muscle bundles, which sometimes affect nerve fibers. Additionally, the PRO–UFAs’ gel treated group recorded slight improvement coupled with marked neuritis that exhibited numerous inflammatory cells’ infiltration and vacuolated and demyelinated fibers. Moreover, excessive perineuritis with adjacent muscle bundles necrosis and accumulation of eosinophilic and karyorrhectic tissue debris were recognized. In contrast, PRO–CTS–UFAs gel showed a significant nerve improvement in longitudinal section investigation, apparently with normal myelinated nerve fibers in several examined sections coupled with a few mildly dilated endoneurial blood vessels associated with mild inflammation in the perineuronal tissue (Figure 8).

Kamisli et al. [169] revealed histopathological changes in rats subjected to cisplatin, shrinkage of the cytoplasm, and extensively dark pyknotic nuclei in neurons of the cerebral cortex tissue. In addition, Abdelsameea et al. [15] showed the presence of significant demyelination coupled with Wallerian degeneration of nerve fibers resulting in congestion. Moreover, propranolol also demonstrates prophylactic therapy for joint pain restoring the histological structure to reflect the low-grade severity of inflammation [170]. Furthermore, minimal confluent necrosis and edema are seen in propranolol (10 mg kg^−1^) treated rats with myocardial infarction [171]. In addition, Esmaeeli et al. [172] revealed that propranolol administration decreased the harmful effects of cisplatin on radiotracer uptake, histological manifestations that may provide potential benefits in the cisplatin nephrotoxicity model. Furthermore, arthritic rat sections treated with non-selective β-blocker carvedilol exhibited relatively small region damage to the articular surface, thicker articular cartilage, subchondral bone, and a degree of hypercellularity and cloning [48]. Based on previous research, propranolol potentiates the adrenergic receptor blockade, linked to possible histopathological changes in different animal models.

##### Immunohistochemical Staining

**Figure 9:**
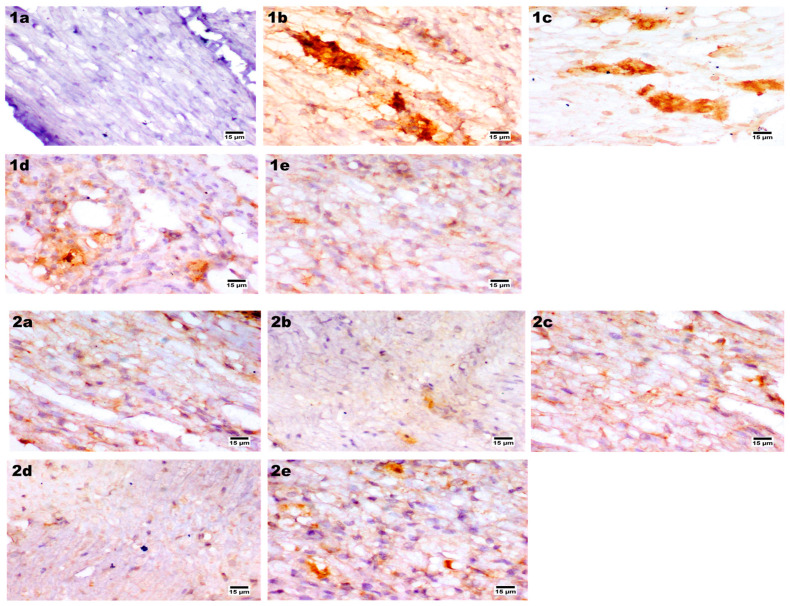

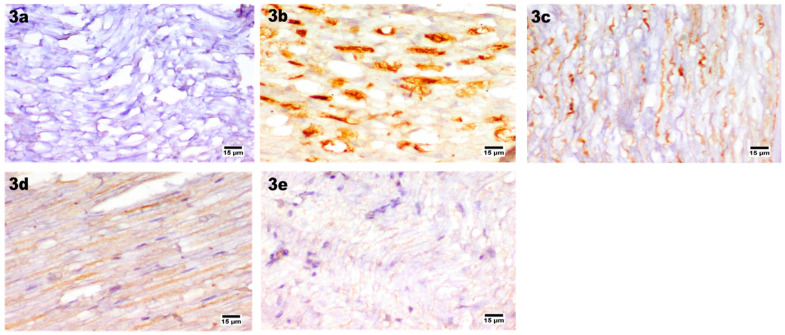
Photomicrographs of rat sciatic nerve sections (immunostained; 15×) for BCL-2 associated x showing the effect of 30-day daily dorsal application administration of PRO solution, PRO–UFAs gel, and PRO–CTS–UFAs gel against cisplatin-induced sciatic nerve injury. (**1a**) A normal control rat section showing normal showing negative expression of BCL-2 associated x; (**1b**) A cisplatin positive control rat section showing strong positive expression; additionally, both (**1c**) PRO solution and (**1d**) PRO–UFAs gel group showing moderate expression BCL-2 associated x; in contrast (**1e**) PRO–CTS–UFAs gel investigation revealed a mild expression. Furthermore, immunostaining myelin basic protein (MBP) revealed that (**2a**) MBP normal control group showed normal expression. Meanwhile, marked decreased expression was detected in nerve fibers of cisplatin positive control group (**2b**). Moderate expression was detected in PRO solution and PRO–UFAs gel (**2c**,**d**), while enhanced expression of MBP was observed for the PRO–CTS–UFAs gel treated group (**2e**). Additionally, TNF α immunostained (**3a**) normal control group showed an absence of its expression in sciatic nerve fibers; (**3b**) the cisplatin positive control group reveals a strong positive expression for TNF α in nerve fibers. While (**3c**,**d**) moderate expression was detected in PRO solution and PRO–UFAs gel, (**3e**) limited expression of TNF-α was observed in PRO–CTS–UFAs gel.

The radiation-induced neuropathy of the sciatic nerve has reported the presence of tissue apoptosis and confirmed dysregulation of Bcl-2 and Bax expression in sciatic nerve tissue [173,174,175]. In addition, the cisplatin-resistant ovarian cancer cell line model also exhibited apoptosis via intrinsic and extrinsic mechanisms incorporating p53 alterations [176]. Bcl-2 family proteins’ expression abnormality and upregulation of apoptosis inhibitors that block the effect of caspases and stabilize the mitochondrial permeability pore [177,178]. Our study evaluated the apoptotic effect on sciatic nerve BCL-2 associated x immunohistochemical examination for the normal control group that showed negative expression of BCL-2 in sciatic nerve fibers. In contrast, cisplatin positive control one showed a strong positive expression in nerve fibers. PRO–CTS–UFAs gel revealed a mild expression of BCL-2 associated x, while PRO solution and PRO–UFAs gel showed a moderate expression (Figure 9). In agreement, previous studies reported that propranolol significantly downregulates B cell lymphoma-2 and BCl-2 associated X protein, which may be related to the TLR4/NF-κB (p65) signal in isoproterenol-induced myocardial fibrosis in mice [179,180,181]. Recently, studies demonstrated that propranolol revealed significant suppression of p38 protein expression that primarily regulates cell proliferation, migration, cell differentiation, and BCL-2 family, inhibiting apoptosis [182,183]. In addition, BCl-2 is considered a tissue homeostasis indicator in vascular, heart, and neurodegenerative diseases [184,185]. Additionally, several studies reported the role of hyperglycemia-induced peripheral neuropathy associated with decreased BCl-2, increased Bax, cleaved caspase-3, and cell apoptosis [186,187,188].

The myelin basic protein (MBP) is a critical regulatory protein for the myelination of nerves, as it maintains the myelin structure and membrane lipid interaction [189,190]. It is an indicator that reflects the amount of myelin and its expression level in myelin sheath damage [191], plays a critical role in supporting neuronal functions [192,193]. Our experimental results illustrated that the MBP control group showed normal expression in sciatic nerve fibers in contrast with the cisplatin positive control group, which showed a remarkable decrease in MBP expression in nerve fibers. Enhanced expression of MBP was observed for the PRO–CTS–UFAs gel treated group, while moderate expression was detected in PRO solution and PRO–UFAs gel (Figure 9).

Previous data indicated that cisplatin-induced sciatic nerve demonstrated heat hypoalgesia that induced demyelination with delayed impulse conduction and MBP expression [194,195]. Additionally, attributed apoptosis of Schwann cells (SC) is present by cisplatin administration [196,197]. The spinal cord microglia model demonstrated that propranolol treatment decreases interleukins’ production and frequency of spinal cord Th17 cells, enhancing MBP expression [55,198]. Furthermore, propranolol improved functional disability, tremors, and ataxia in multiple sclerosis, restoring MBP [199,200]. All these reports confirmed our results that propranolol administration along with cisplatin protects rats’ sensory and motor neuropathy, evidenced by enhancement of MBP expression.

Additionally, cisplatin induced neurotoxicity with a severe inflammatory and proinflammatory mediator’s induction, such as TNF α and NF-κB [201,202]. TNF-α is an essential mediator of chronic inflammation and a significant contributor to peripheral nerve injury and neuropathic pain [203,204]. In PNI, endogenous TNF-α is immediately released by resident cells, such as Schwann cells and macrophages, leading to elevated levels of TNF-α at the site of injury [205,206]. Our results observed that TNF α was not expressed in sciatic nerve fibers of the normal control group. In contrast, the cisplatin positive control group revealed a strong positive expression for TNF α in nerve fibers. Limited expression of TNF-α was observed in PRO–CTS–UFAs gel, while moderate expression was detected in PRO solution and PRO–UFAs gel (Figure 9).

Previous studies reported that in the CCI-induced neuropathy model, the TNF-α, IL-1β signals, and infiltration of CD68+ inflammatory cells induced a partial decrease after nerve release [207,208]. Our study showed that TNF-α signaling was an essential feature of PNS autoimmunity [209], since TNF-α expression limitation protected against PIN [210,211]. However, activation of TNF-α triggers many proteins involved in apoptosis [212,213]. Additionally, treatment with non-selective adrenergic antagonist carvedilol suppresses pro-inflammatory cytokines TNF-α in a complete Freund’s adjuvant-induced rat rheumatoid arthritis model [48,214,215]. In addition, propranolol exhibits adjuvant activity in the breast cancer vaccine model by modulating cytokines and TNF-α [216,217]. Propranolol was also reported to control acute ischemic stroke patients with lymphopenia through its role in enhancing TNF-α, IL-10, and TNF-α/IL-10 [218,219]. Furthermore, it was reported to protect dopaminergic neurons in rats with experimentally induced parkinsonism TNF-α production suppression [220,221]. According to De Arajo Jnior et al. [222], carvedilol could suppress the production of TNF- and other cytokines in a peritonitis animal model. This was consistent with previous research demonstrating propranolol’s anti-inflammatory potential in various animal models, which was mediated by suppression of pro-inflammatory cytokines such as TNF-α [223,224]. Previous studies have speculated that propranolol exerts therapeutic effects on neuropathic pain-related pathogenesis based on its immunomodulatory, anti-inflammatory, and antioxidant potentials.

## 4. Conclusions

This is the first trial to use CTS-coated UFAs hydrogel loaded with PRO as a bioactive scaffold for treating PNI in a rat model. The optimized nanoparticles were 336.12 nm in size, had a surface charge of 65.24 mV, 85.32 percent entrapment, and were highly stable. PRO–CTS–UFAs had better permeability and a longer release time in ex vivo permeability and release experiments. In vivo experiments revealed that the PRO–CTS–UFAs-treated group had significantly lower MDA levels, as well as lower peripheral myelin 22 gene expression; however, CAT and GSH levels were elevated. Furthermore, histopathological examination investigated normal myelinated nerve fibers with mild inflammation. In addition, immune staining sections represented MBP re-expression, BCL-2 mild expression, and absence of TNF-α expression. Our research presented a novel opportunity for the efficient delivery of PRO via CTS–UFAs assembly, which may be beneficial for treating cisplatin-induced sciatic nerve damage. However, additional pharmacokinetic studies on suitable animal models should be conducted to demonstrate the superiority and safety of the customized PRO–CTS–UFA over conventional medications.

## Figures and Tables

**Table 1:** Full factorial design 2^4^ was used for optimization of PRO–UFAs formulation.

Factors	Levels	
	Low (−1)	High (+1)
Independent variables		
A = Span type	Span 20	Span 60
B = Oleic acid amount (mg)	20	40
C = Cholesterol amount (mg)	15	30
D = Sonication time (min)	0	15
Responses (dependent variables)	Desirability constraints	
Y_1_ = EE%	Maximize	
Y_2_ = PS (nm)	Minimize	
Y_3_ = PDI	˂0.5	
Y_4_ = ZP (mV)	Minimize	
Y_5_ = Q6h (%)	Maximize	

EE%: entrapment efficiency percent, PS: particle size, PDI: polydispersity Index, ZP: zeta potential, Q6h: cumulative release after 6 h.

**Table 2:** Experimental runs, independent variables, and measured responses of the 2^4^ full factorial experimental designs of PRO–UFAs.

Ufasomes Formulation	A	B	C	D	Y1	Y2	Y3	Y4	Y5
	Span Type	Oleic Acid Amount (mg)	Cholesterol Amount (mg)	Sonication Time (min)	EE%	PS (nm)	PDI	ZP (mV)	Q6h %
U1	span 20	40	15	15	64.72 ± 1.12	402.62 ± 25.01	0.28 ± 0.023	−69.32 ± 2.25	81.31 ± 2.33
U2	span 60	20	15	0	86.84 ± 2.04	432.13 ± 15.66	0.62 ± 0.012	−33.52 ± 1.23	68.55 ± 1.12
U3	span 20	40	15	0	77.30 ± 1.52	302.78 ± 18.52	0.32 ± 0.016	−67.30 ± 1.11	77.28 ± 1.65
U4	span 60	40	30	0	96.13 ± 3.22	510.44 ± 20.32	0.61 ± 0.021	−33.95 ± 1.06	49.61 ± 0.98
U5	span 20	40	30	0	91.22 ± 2.67	326.85 ± 17.65	0.56 ± 0.015	−62.60 ± 1.14	53.62 ± 2.24
U6	span 20	20	30	0	88.32 ± 1.65	385.80 ± 20.31	0.59 ± 0.032	−34.47 ± 2.50	71.30 ± 3.23
U7	span 20	20	15	0	74.97 ± 2.66	408.29 ± 14.82	0.36 ± 0.022	−64.27 ± 2.14	83.21 ± 4.87
U8	span 20	40	30	15	90.44 ± 3.91	397.57 ± 22.54	0.44 ± 0.032	−66.24 ± 2.34	61.29 ± 2.45
U9	span 60	20	15	15	78.67 ± 2.54	351.11 ± 23.70	0.52 ± 0.034	−39.55 ± 3.01	74.88 ± 3.21
U10	span 60	20	30	0	90.47 ± 3.43	480.26 ± 24.13	0.71 ± 0.016	−31.15 ± 1.13	59.57 ± 2.05
U11	span 20	20	15	15	53.64 ± 1.04	430.46 ± 26.23	0.31 ± 0.034	−71.12 ± 1.45	87.75 ± 3.98
U12	span 60	40	15	0	87.61 ± 2.21	470.42 ± 15.74	0.59 ± 0.010	−36.87 ± 1.08	65.58 ± 4.11
U13	span 60	40	15	15	79.45 ± 2.34	490.21 ± 10.74	0.46 ± 0.03	−40.36 ± 2.23	79.63 ± 4.56
U14	span 60	40	30	15	97.52 ± 4.05	485.86 ± 18.00	0.53 ± 0.016	−37.41 ± 1.65	56.35 ± 1.34
U15	span 60	20	30	15	82.93 ± 2.76	356.51 ± 11.65	0.57 ± 0.021	−43.87 ± 1.45	70.88 ± 2.12
U16	span 20	20	30	15	79.89 ± 1.94	382.23 ± 10.43	0.42 ± 0.035	−43.59 ± 2.06	80.44 ± 7.55

EE%: entrapment efficiency percent, PS: particle size, PDI: polydispersity index, ZP: zeta potential, Q6h: cumulative release after 6 h (%). Data are mean values (*n* = 3) ± SD.

**Table 3:** Primers sequence of peripheral myelin 22 gene.

	Forward Sequence	Reverse Sequence
**Peripheral myelin 22**	CTCCTCGCAGGCAGAAACTC	TGGCCAGCTCTCCTAAC
**GAPDH**	TGGATTTGGACGCATTGGTC	TTTGCACTGGTACGTGTTGAT

**Table 4:** Output data of the 2^4^ factorial analyses of PRO–UFA formulation.

Responses	R^2^	Adjusted R^2^	Predicted R^2^	Adequate Precision	Significant Factors
**EE%**	0.97	0.92	0.74	15.59	A, B, C, D
**PS (nm)**	0.98	0.95	0.83	18.79	A, B
**PDI**	0.98	0.94	0.80	16.76	A, C, D
**ZP (mV)**	0.96	0.87	0.57	10.06	A, B, C
**Q6h (%)**	0.98	0.95	0.85	20.57	A, B, C, D

EE%: entrapment efficiency percent, PS: particle size, PDI: polydispersity index, ZP: zeta potential, Q6h: cumulative release after 6 h (%).

**Table 5:** The experimental values of the optimized PRO–UFAs and PRO–CTS–UFAs (means ± SD, *n* = 3).

Solution	Span Type	Oleic Acid Amount (mg)	Cholesterol Amount (mg)	Sonication Time (min)	EE%	PS (nm)	PDI	ZP (mV)	Q6h %
optimized PRO–UFAs	Span 20	40	22.52	0	82.72 ± 2.33	317.22 ± 6.43	0.441 + 0.02	−62.06 ± 0.07	70.95 ± 8.14
PRO–CTS–UFAs	Span 20	40	22.52	0	85.32 ± 2.65	336.12 ± 4.9	0.445 ± 0.03	65.24 ± 0.10	64.03 ± 1.9

**Table 6:** Ex vivo permeation parameters of PRO-CTS- UFAs and optimized PRO–UFAs versus PRO solution.

Formulation	Lag Time (min)	Jss (µg/cm^2^ h)	Kp (cm/h)	EI
PRO–CTS–UFAs	50.63 ± 2.23	16.98 ± 0.12	0.0169 ± 0.0007	2.45
optimized PRO–UFAs	66.13 ± 4.34	8.12 ± 0.45	0.0082 ± 0.0013	1.19
PRO solution	146.78 ± 10.13	6.91 ± 0.12	0.0069 ± 0.0010	-

Jss: drug flux; Kp: permeability coefficient; EI: enhancement index. Data are mean values (*n* = 3).

## Data Availability

Not applicable.
